# Primary ductal carcinoma of ectopic breast^☆^

**DOI:** 10.1016/j.abd.2021.02.017

**Published:** 2023-01-18

**Authors:** Ariane Sponchiado Assoni, Beatriz Baptista Abreu da Silva, Aline Sponchiado Assoni, Felipe Mauricio Soeiro Sampaio

**Affiliations:** a Department of Dermatology, Hospital Federal de Bonsucesso, Rio de Janeiro, RJ, Brazil; b Private practice, Porto Alegre, RS, Brazil

Dear Editor,

Ectopic breast carcinoma accounts for approximately 0.3% to 0.6% of all breast cancers, with 95% arising from aberrant breast tissue.^1^, ^2^ Clinical diagnosis may be delayed due to the atypical location, similarity to other diseases, and flawed laboratory tests.^3^ This case report describes a patient with primary ductal carcinoma of the ectopic breast in the axilla, diagnosed and treated as hidradenitis suppurativa (HS).

A 62-year-old female patient presented with an erythematous, well-defined nodule measuring 1.5×2.0 cm, with a retracted center, painful and hard on palpation in the left axilla (Fig. 1).

**Figure 1:**
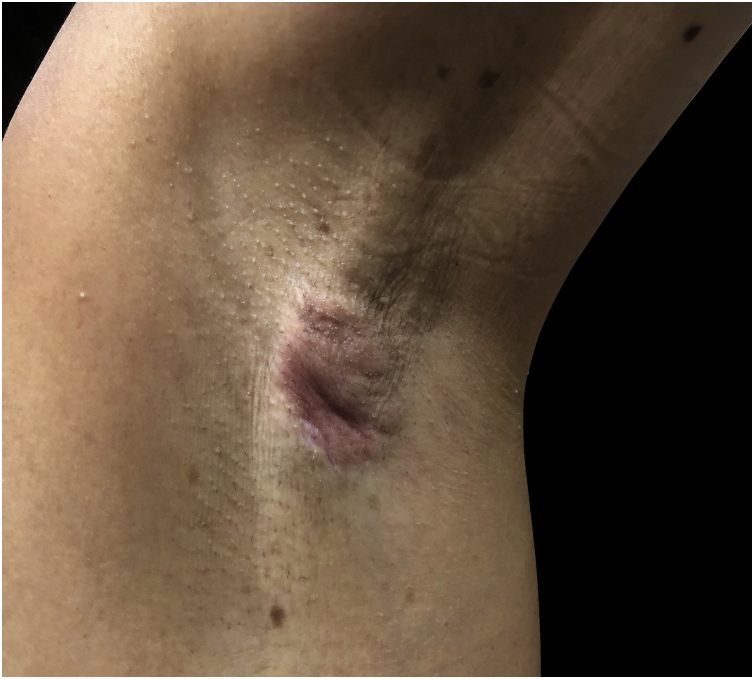
Erythematous, mobile nodule with retraction area.

The clinical diagnosis suggestive of HS was confirmed by ultrasound, which showed an area of ​​subcutaneous hypoechoic thickening and a nodular area. Topical and oral antibiotics were prescribed for 14 days, in addition to intralesional corticosteroid injections, without improvement.

Two 0.5 cm elliptical incisional biopsies were performed and anatomopathological analysis showed preserved epidermis and the presence of rows and clusters of atypical epithelial cells in the dermis (Fig. 2), while the immunohistochemistry showed positivity for pankeratin, suggestive of cutaneous metastasis.

**Figure 2:**
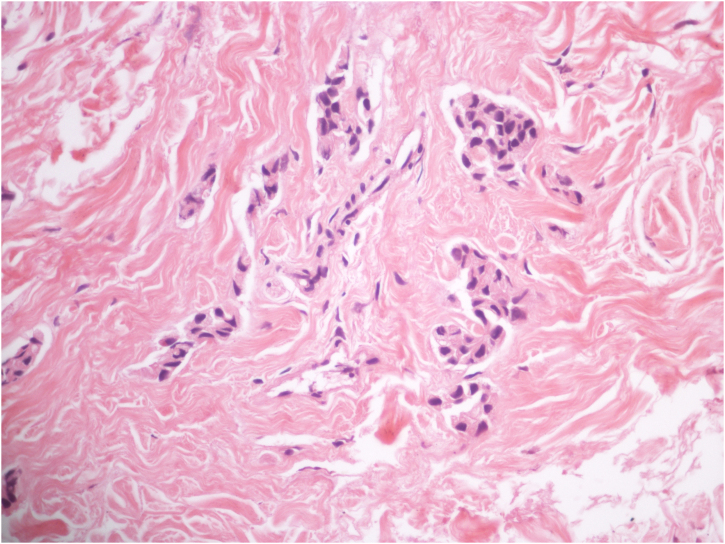
Histopathology of the incisional biopsy showing epithelial aggregates of atypical cells (Hematoxylin & eosin, ×400).

The neoplastic screening did not disclose a primary site. Therefore, total excision of the lesion was performed, which showed dermal infiltration of carcinomatous cells over ectopic mammary gland tissue (Fig. 3). Immunohistochemistry was positive for estrogen and negative for progesterone and human epidermal growth factor receptor-type 2 (HER-2). It was concluded that it was a primary ectopic breast carcinoma with characteristics of invasive ductal carcinoma.

**Figure 3:**
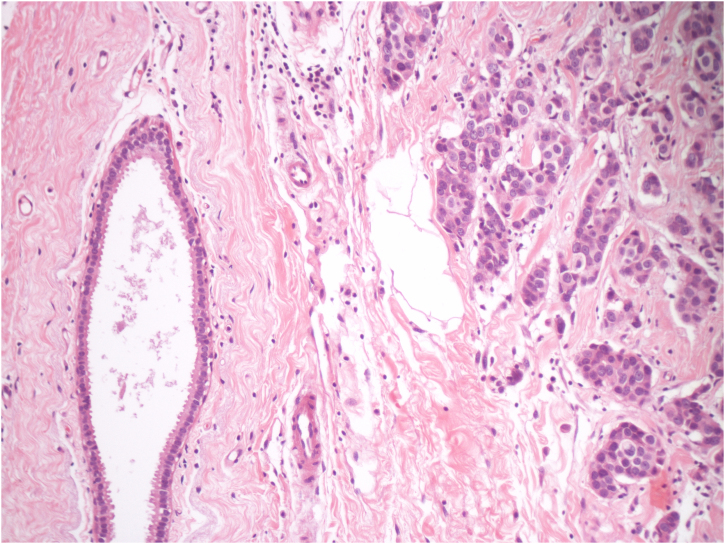
Histopathology of the excisional biopsy showing ectopic mammary glandular tissue and dermal infiltration by carcinomatous cells (Hematoxylin & eosin, ×400).

The patient was referred to the mastology and oncology service, where surgical margins were enlarged and an ipsilateral axillary lymphadenectomy was performed, due to the presence of lymph node metastasis. She underwent complementary treatment with radiotherapy and anastrozole. There has been no recurrence one year on outpatient follow-up and control mammography.

Ectopic breast tissue is subject to the same pathophysiological processes as the topical breast, but malignant changes are more frequent than benign ones.^1^ It can consist of glandular tissue, nipple and areola. It is subdivided into supernumerary breast or aberrant breast tissue. The latter is characterized by the presence of an isolated mammary gland, close to the topical breast and without communication with overlying skin.^1^

Ectopic breast cancer predominates in the female sex. The axillary region is most frequently affected and infiltrating ductal carcinoma accounts for 79% of cases.^1^, ^4^ The most common clinical manifestation is the presence of a unilateral, subcutaneous, irregular, erythematous, indurated nodule showing progressive growth, with or without a nipple and areola.^5^ Ultrasonography is the initial preferential examination, which may show an irregular, hypoechoic, heterogeneous, poorly-defined nodule; the accessory mammary gland may be detected.^5^ The diagnosis is usually a late one, with an average delay of 40 months, which can lead to worse prognosis. The confirmation is achieved with histopathological analysis.^3^, ^5^ The management of ectopic breast carcinoma follows conventional breast cancer treatment and staging. There is no consensus on the prophylactic excision of the ectopic breast tissue.^3^, ^5^

Although it is a rare condition, it is essential to recognize it and consider tumors along the milk line. It is important to emphasize the importance of the histopathological analysis, even in the presence of a probable benign lesion.

## Financial support

None declared.

## Authors’ contributions

Ariane Sponchiado Assoni: Design and planning of the study; drafting and editing of the manuscript; critical review of the manuscript.

Beatriz Baptista Abreu da Silva: Design and planning of the study; drafting and editing of the manuscript; critical review of the literature.

Aline Sponchiado Assoni: Drafting and editing of the manuscript; critical review of the literature.

Felipe Mauricio Soeiro Sampaio: Approval of the final version of the manuscript; effective participation in research orientation; critical review of the manuscript.

## Conflicts of interest

None declared.
